# Differential Requirement for *SUB1* in Chromosomal and Plasmid Double-Strand DNA Break Repair

**DOI:** 10.1371/journal.pone.0058015

**Published:** 2013-03-12

**Authors:** Lijian Yu, Michael R. Volkert

**Affiliations:** Microbiology and Physiological Systems, University of Massachusetts Medical School, Worcester, Massachusetts, United States of America; National Cancer Institute, United States of America

## Abstract

Non homologous end joining (NHEJ) is an important process that repairs double strand DNA breaks (DSBs) in eukaryotic cells. Cells defective in NHEJ are unable to join chromosomal breaks. Two different NHEJ assays are typically used to determine the efficiency of NHEJ. One requires NHEJ of linearized plasmid DNA transformed into the test organism; the other requires NHEJ of a single chromosomal break induced either by HO endonuclease or the I-SceI restriction enzyme. These two assays are generally considered equivalent and rely on the same set of NHEJ genes. PC4 is an abundant DNA binding protein that has been suggested to stimulate NHEJ. Here we tested the role of PC4's yeast homolog *SUB1* in repair of DNA double strand breaks using different assays. We found *SUB1* is required for NHEJ repair of DSBs in plasmid DNA, but not in chromosomal DNA. Our results suggest that these two assays, while similar are not equivalent and that repair of plasmid DNA requires additional factor(s) that are not required for NHEJ repair of chromosomal double-strand DNA breaks. Possible roles for Sub1 proteins in NHEJ of plasmid DNA are discussed.

## Introduction

In cells, double strand DNA breaks (DSBs) can be induced by ionizing radiation, oxidation, DNA damaging chemicals, replication errors and others [1], [2]. Because a single DSB can cause chromosomal loss or translocation which may lead to cell death or cancerous transformation, repair pathways for DSBs are conserved in eukaryotes ranging from yeast to human and have been the focus of numerous studies [2], [3]. DSBs can be repaired by two distinct repair pathways: homology-directed repair (HR) and non homologous end joining (NHEJ) [4], [5]. HR is a repair process that finds homologous sequences in the genome and uses it as a template to repair the chromosomal break [6]–[8]. In contrast, NHEJ is a repair process that brings the ends of the broken DNA together and repairs the break by direct ligation [9]–[11]. NHEJ requires the KU complex, DNA ligase and other factors [11], [12]. Since the DSB ends joined in NHEJ may result in alterations of DNA bases at the junction, NHEJ is considered to be more error-prone [13].

Two experimental approaches have been widely used to introduce DSBs into cells to study the NHEJ repair pathway [11], [14]–[16]. The first approach is to induce DSBs in chromosomes *in vivo*. This can be achieved by treating cells with ionizing radiation or chemicals that generate DSBs randomly throughout the genome. Alternatively, meganucleases such as HO and I-SceI endonucleases have been used to induce site specific DSBs in the chromosome [14], [17]. Because induction of the meganucleases can be controlled and the genomic location of the DSB can be precisely engineered, many details of the molecular events in DSB repair have been obtained using this approach [18]–[20]. The other approach used to study NHEJ is to introduce DNA containing a specific break into the cells. This is usually achieved by linearizing a plasmid using a restriction enzyme and transforming the cells with the linearized DNA [21]. Because the sequence flanking the DSB has no homology in the host genome, cells can only maintain the plasmid by first repairing it. Therefore the efficiency of NHEJ repair can be determined by the transformation efficiency of the linear plasmid relative to that of the control circular plasmid. The plasmid repair assay has proven useful by studies that characterized several key NHEJ factors [21]–[23]. It is noteworthy that both approaches have been used to study NHEJ in human and yeast cells [24]–[31].

PC4 is a small nuclear protein produced by the human SUB1 gene. It can bind to double strand DNA and single strand DNA without sequence specificity [32], [33]. It was first isolated as a transcription coactivator in 1994, able to promote basal transcription at low concentrations [34], [35]. The yeast *SUB1* gene is homologous to PC4 in sequence and activity. Both proteins bind DNA and function as transcription coactivators [36]–[39]. Additionally, Wang *et al* found that PC4 and its yeast homolog *SUB1* suppress oxidative mutagenesis and confer oxidative resistance in yeast, suggesting a role for PC4 in DNA repair or DNA damage prevention [40]. Interestingly, Batta *et al* showed that PC4 promotes DNA ligation *in vitro* and suggested that it may stimulate repair of DNA breaks by NHEJ [41]. However, it was less certain if PC4 is required for NHEJ *in vivo*. In the present study we tested the role of PC4's yeast homolog *SUB1* in NHEJ *in vivo* by using several different assays; HO-induced chromosomal breaks, I-SceI induced chromosomal breaks and restriction enzyme induced breaks in plasmid DNA prior to transformation. NHEJ was then assayed comparing yeast *sub1* mutants with wild type and *yku70* mutant cells. Unexpectedly, the yeast *sub1Δ* mutant displays differential repair capacities for DNA breaks in chromosomes and in linearized plasmids, showing a severe defect in the repair of transformed, linearized plasmid DNA breaks, while being fully capable of repairing chromosomal DNA breaks.

## Materials and Methods

### Yeast strains

Yeast strains used in this study are listed in Table 1. The PCR based gene replacement method was used to create the yeast knock out strains [42], [43].

**Table 1:** Yeast strains used in this study.

Strain	Original name, genotype (annotation)	Reference
MVY101	FY833, Mata *ura3-52 leu2Δ1 trp1Δ63 his3Δ200 lys2Δ202*	[57]
MVY105	MVY101 with *sub1Δ*::hisG	[57]
MVY601	MVY101 with *yku70Δ*::*HIS3*	this study
MVY603	MVY101 with *sub1Δ*::hisG *yku70Δ*::*HIS3*	this study
MVY610	JKM179, *MAT*α *hoΔ hmlΔ*::*ADE1 hmrΔ*::*ADE1 ade1-100 leu2,3-112 lys5 trp1Δ*::hisG *ura3-52 ade3*::*GAL-HO*	[52]
MVY614	MVY610 with *yku70Δ::URA3*	[52]
MVY617	MVY610 with *sub1Δ*::*TRP1*	this study
MVY625	MVY610 with *yku70Δ::URA3 sub1Δ*::*TRP1*	this study
MVY665	YW714, *MAT*α, *ade2::SD2-*::*URA3 his3D1 Leu2D0 LYS2 MET15 ura3D0*	[48]
MVY666	YW713, *MAT*α, *ade2::SD2-::URA3 his3D1 Leu2D0 LYS2 MET15 ura3D0 yku70::kanMX4*	[48]
MVY667	MVY665 with *sub1::KanMX4*	this study
MVY696	MVY665 with *sub1::KanMX4 yku70::HIS3*	this study

### Transformation

The yeast transformation procedure is as described by Knop et al. [44]. Briefly, cells are grown to early log phase, sonicated briefly, made transformation-competent and used immediately or stored at −80°C. 100–200 ng DNA is used in each transformation. After transformation, cells are plated directly onto minimal media lacking the selected nutrient or incubated in YPD medium (1% yeast extract, 2% peptone, 2% glucose) for 2 hours before plating onto YPD containing 200 µg/ml G418 for KanMX selection.

### Plasmid NHEJ repair assay

The plasmid repair assay is similar to the procedures described elsewhere [21], [45]. Linearized plasmid DNA is produced by digesting the plasmid with the specified restriction enzyme, followed by gel-purification of linear DNA. Both linearized and circular plasmids are used to transform the yeast cells. Colonies are counted 3–4 days after transformation. The plasmid repair efficiency is calculated as the transformation efficiency of the linearized plasmid divided by the transformation efficiency of the circular plasmid.

To quantify the ratio of mutagenic ligation events, yeast cells are transformed with NcoI-linearized pMV1328, which cuts within the *KanMX6* coding sequence, then selected on plates lacking leucine. After incubation, Leu^+^ transformants are streaked on YPD agar medium containing 200 µg/ml G418 to test *KanMX* function. Colonies that are Leu+but G418-sensitive are counted as mutagenic ligation events.

### HO induction and cell survival

Wild type and the mutant cells are incubated in YEP-raffinose (1% yeast extract, 2% peptone, 2% raffinose) to log phase (OD_600_<0.5) and sonicated briefly. Half of each culture is supplemented with 2% galactose to induce the HO endonuclease for the indicated times. Both the induced and uninduced cells are diluted in water and plated onto YPD agar medium to count the number of viable cells. The presence of glucose in the YPD medium suppresses HO expression.

The induction of DSBs is measured by the PCR based method as described [46]. Briefly, at the indicated times after adding 2% galactose to the cell culture, an aliquot of cells is removed and genomic DNA is extracted using the phenol and glass beads method [47]. Real time PCR is performed on a ViiA7 QPCR machine using the primers SG2285 (AATATGGGACTACTTCGCGCAACA) and SG2286 (CGTCACCACGTACTTCAGCATAA) to amplify *MAT*α, which contains the HO site, and the primers SG525 (TTGGATTTGGCTAAGCGTAATC) and SG526 (CTCCAATGTCCCTCAAAATTTCT) to amplify the *SMC2* control PCR products as described [46]. The decline in the ratio between the PCR products of *MAT*α and *SMC2* represents the increase in the number of the cells with a DSB in the *MAT*α locus. DSB induction is normalized to the respective 0 time point.

### Suicide deletion assay

The suicide deletion assay is performed as described in Karathanasis and Wilson [48]. Briefly, wild type and mutant cells are incubated in medium lacking uracil for 3 days to reach stationary phase and keep the *I-SceI-Ura3* insert under selection. Cells are then sonicated briefly and diluted in water. Dilutions are plated on synthetic, complete agar medium with uracil to allow loss of the insert and galactose as the carbon source (SC-galactose) to induce I-SceI. After I-SceI induction, survival requires loss of the insert and repair of the DSB. Surviving colonies growing on SC-galactose exhibit a white color if the *ADE2* gene is restored by NHEJ repair, or a red color if the repair is inaccurate and the *ADE2* gene is disrupted. The frequency of mutagenic ligation is the number of red colonies divided by the total number of colonies on the SC-galactose plates.

### Statistics

All experiments were performed at least three times (n>3). Error bars represent standard errors. Two tailed student t-test is used to calculate the P values.

## Results

### Reduced plasmid repair in the *sub1Δ* mutant

**Figure 1:**
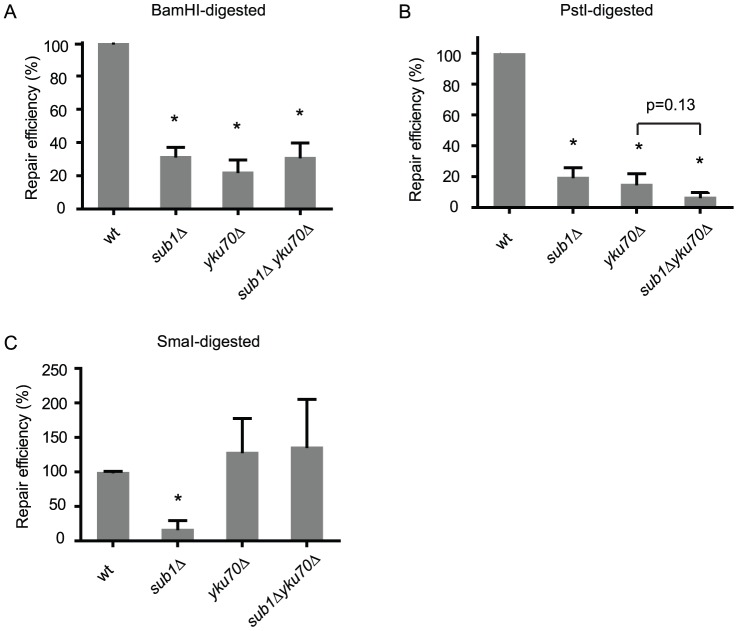
*SUB1* is required for repair of dsDNA breaks in plasmid DNA. Repair efficiency is the ratio of the number of stable transformants obtained when transformed with linearized versus circular pRS315 plasmid DNA. Data are normalized to the ligation efficiency of wild type. Asterisks * indicate a significant difference from wild type (P<0.05). All strains are derivatives of the wild type (MVY101), *sub1*Δ (MVY105), *yku70* (MVY601), *sub1*Δ *yku70* (MVY603). **A**. Repair efficiencies of the BamHI-linearized plasmid. BamHI produces a unique DSB in pRS315 with 5' end overhangs. **B**. Repair efficiencies of the PstI-linearized plasmid. PstI produces a unique DSB in pRS315 with 3' end overhangs. **C**. Repair efficiencies of the SmaI-linearized plasmid. SmaI produces a unique DSB in pRS315 with blunt DNA ends.

PC4 has been shown to stimulate the ligation of DNA *in vitro*, suggesting that PC4 plays a role in NHEJ [41]. Here we tested if the yeast homolog *SUB1* is required for NHEJ in yeast. We first used the plasmid repair assay to test if the *sub1* mutant is impaired to rejoin a double strand DNA break that is introduced into the plasmid by restriction digestion and transformed into the cell [21]. BamHI linearizes plasmid pRS315 with 5' sticky ends in a region that has no homology in the yeast genome. We found that the *sub1Δ* mutant exhibits significantly reduced repair capacity, comparable to the level seen in the *yku70Δ* mutant, which is known to be deficient in NHEJ (Figure 1A). The *sub1Δ yku70Δ* double mutant does not exhibit a further reduction in NHEJ efficiency, indicating that Sub1 functions in the KU dependent repair pathway.

Next we tested if *SUB1* is required for repair of DNA breaks with different DNA end structures. We used the restriction enzyme PstI to generate DSB in pRS315 with 3' sticky ends. Similar to the BamHI-generated DSB, the *sub1Δ* mutant has a reduced repair efficiency (Figure 1B). The *sub1Δ yku70Δ* double mutant does not have a significant further reduction in repair efficiency (P = 0.13), supporting previous results that Sub1 and Yku70 function in the same NHEJ pathway.

DSBs with blunt ends may be repaired by NHEJ in a KU independent fashion with low efficiencies [21], [49]–[51]. We used SmaI to generate a blunt-end DSB in pRS315. Surprisingly, the *sub1Δ* mutant exhibits a reduced repair efficiency for blunt-end DSBs, while the *yku70Δ* mutant is highly proficient (Figure 1C). This suggests that Sub1 is required for repair of blunt-end DSBs but not Yku70. The *sub1Δ yku70Δ* double mutant has a repair capacity similar to the *yku70Δ* mutant, raising the possibility that repair of blunt-end DSBs is channeled into the Yku70 independent pathway. In this pathway Sub1 is dispensable, or alternatively Yku70 may inhibit blunt end ligations in the absence of Sub1.

**Figure 2:**
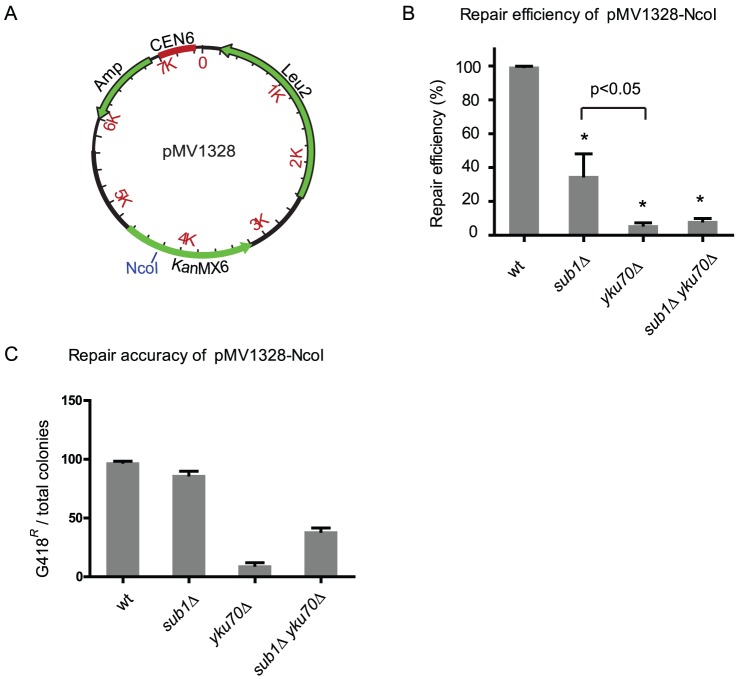
Ligation accuracy of the plasmid repair . Strains, same as in Figure 1. **A.** Plasmid map of pMV1328. The unique NcoI restriction site within the *KanMX6* gene is indicated. **B.** Repair efficiencies of the mutants when the NcoI linearized pMV1328 is used in the repair assay. Asterisks * indicate a significant difference from wild type (P<0.05). **C.** Accuracy of plasmid ligation is measured by testing Leu+transformants for G418 resistance (G418^R^), which requires accurate religation of the NcoI site within the *KanMX6* gene.

When NHEJ efficiency is reduced, the remaining ligation events can become mutagenic, as is the case in the KU mutant *yku70* [21]. To test if the residual NHEJ seen in the *sub?* mutant is mutagenic, we inserted the *KanMX6* gene into pRS315 to make the plasmid pMV1328 (Figure 2A). When the NcoI restriction enzyme is used to cut the single NcoI site that lies within the *KanMX6* gene, a functional *KanMX6* gene will be inherited only if NHEJ is accurate. This can be detected by testing Leu^+^transformants for resistance to G418. We first examined the mutants for their abilities to repair the NcoI-linearized pMV1328. As shown in Figure 2B, the *sub?* mutant has a reduced repair efficiency compared to wild type, confirming our previous results obtained with plasmid pRS315. However, the *yku70Δ* mutant exhibits a lower repair efficiency than the *sub1Δ* mutant (P<0.05), indicating that the DNA sequence flanking the DNA break may affect the efficiency of NHEJ [50]. Nonetheless, the *sub1Δ yku70Δ* double mutant does not exhibit an additive deficiency, suggesting Sub1's role in NHEJ depends on Yku70. Interestingly, most cells that repair the NcoI-cut in pMV1328 are resistant to G418 in both wild type and the *sub1Δ* mutant (Figure 2C), suggesting that NHEJ in the *sub1Δ* mutant is highly accurate as is the case in wild type cells. In contrast, repair in the *yku70Δ* mutant is highly mutagenic (Figure 2C) as previously reported [21]. This suggests Sub1 and Yku70 may have different roles in the same NHEJ pathway. More puzzling, the double mutant exhibits a higher repair accuracy than the *yku70Δ* mutant, but lower than the *sub1Δ* mutant. This raises the possibility that the fidelity of NHEJ results from the interplay of multiple factors and the mutagenicity of the *yku70* mutant can be partially masked by depleting Sub1.

### Efficient joining of chromosomal breaks in the *sub1Δ* mutant

**Figure 3:**
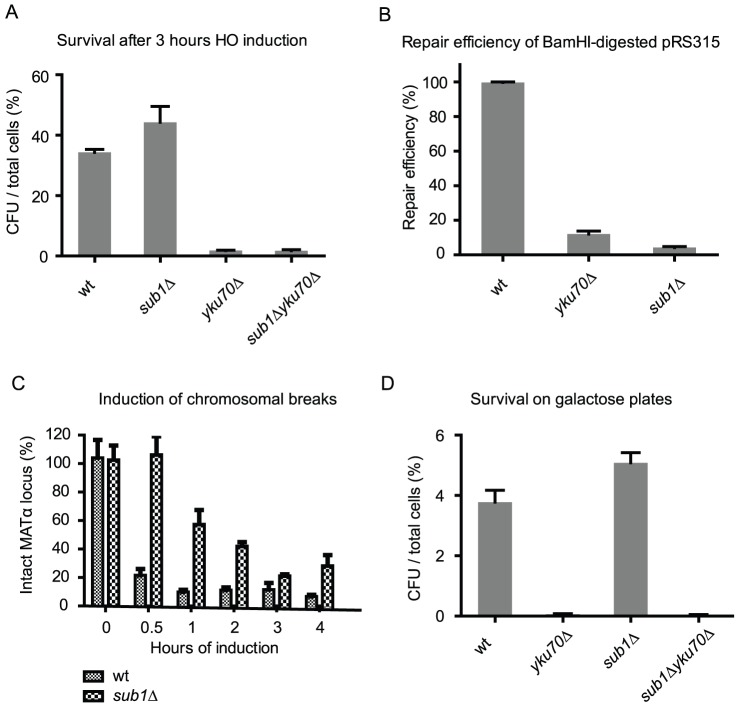
*SUB1* is not required for repair of chromosomal breaks. All strains used are derived from the ‘donorless’ strain JKM179 (MVY610): *sub1*Δ (MVY617), *yku70* (MVY614), *sub1*Δ *yku70* (MVY625). CFU: Colony Forming Units. **A**. Cell survival after HO induction for 3 hours followed by repression by glucose. **B**. Plasmid repair assay is performed in the JKM179 background. Repair efficiencies are normalized to wild type. **C**. Quantification of DSBs after HO induction. The yeast *MAT*α locus is cleaved by HO endonuclease and the fraction of remaining, intact *MAT*α DNA is determined by real time PCR. **D**. Cell survival when HO is constantly induced by galactose. Cells are plated on galactose-containing plates to count surviving cells and glucose-containing plates to count the total number of cells.

Our results suggest that Sub1 is important for repair of DSBs in transformed plasmid DNA. We next asked if Sub1 is required for repair of chromosomal breaks. We first used the inducible HO endonuclease system to test if the *sub1Δ* mutant can repair the induced chromosomal breaks efficiently. After transcriptional induction of the HO endonuclease a single chromosomal break is produced in the cell. After HO expression is again repressed, cells that successfully repair the DNA break will survive. We used the strain JKM179, which lacks the homologous sequences needed to repair HO-induced breaks by HR, and can only use NHEJ to carry out the repair needed for survival [52]. Unexpectedly, as shown in Figure 3A, the *sub1Δ* mutant survives the HO induced chromosomal breaks even better than wild type while the *yku70Δ* mutant and the *sub1Δyku70Δ* double mutant succumb to the induced chromosomal breaks.

We tested if the *sub1Δ* mutant in the JKM179 background is also deficient in repair the plasmid-borne DSB. Results in figure 3B show that NHEJ repair in the *sub1Δ* mutant and the *yku70Δ* mutant is greatly reduced, suggesting that the NHEJ deficiency in the *sub1Δ* mutant in repairing plasmid DSB is not restricted to one yeast strain background.

Sub1 has been found as a transcription cofactor that can activate basal transcription [34], [35]. Therefore we tested if HO induction is compromised in the *sub1Δ* mutant. Quantitative PCR analysis of DSB induction [46] showed that the *sub1Δ* mutant does have fewer DSBs after HO induction (Figure 3C). However, 3 hours after HO induction, the difference between wild type and the *sub1Δ* mutant is only 10%. After subtracting this difference from the survival ratio in Figure 3A, the *sub1* mutant appears not to have reduced survival after chromosomal breaks are induced.

Furthermore, we plated the mutants directly on galactose plates to continuously express the HO nuclease. In the constant presence of HO nuclease, the cycles of break production and repair will continue until, either HO induction is lost, or the HO recognition site is mutated during repair. As shown in Figure 3D, the continuously induced chromosomal break is highly lethal to the *yku70Δ* mutant. However, the *sub1Δ* mutant does not exhibit reduced survival, suggesting chromosomal breaks are repaired efficiently by NHEJ in the *sub1Δ* mutant. The double mutant has a survival rate similar to the *yku70Δ* mutant, further indicating a dispensable role for Sub1 in repairing chromosomal breaks.

**Figure 4:**
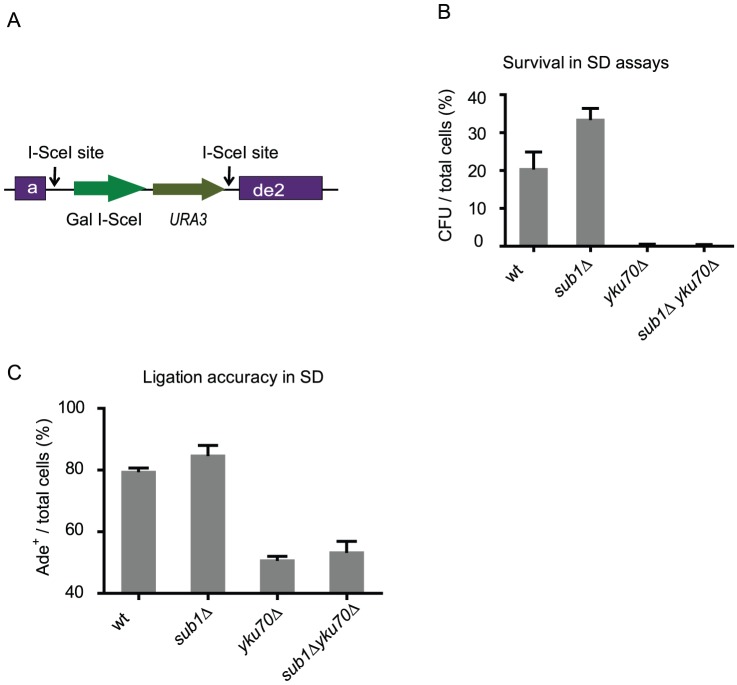
Sub1 is not required for maintaining repair accuracy of chromosomal breaks. Strain used: wild type (MVY665), *yku70Δ* (MVY666), *sub1Δ* (MVY667), *sub1Δ yku70Δ* (MVY696). **A**. Schematic drawing of the suicide deletion system. The *I-SceI* gene, which is expressed from a Gal-inducible promoter, and the URA3 gene with its own promoter are inserted between two I-SceI sites in the *ADE2* gene [48]. After Gal induction, the DNA fragment containing *I-SceI* and *URA3* is lost and the functional *ADE2* gene is restored only if NHEJ is accurate. **B**. Percent of surviving cells when cells are plated on galactose-containing plates. Total number of cells is determined by plating cells on YPD plates. CFU: Colony Forming Units. **C**. Ligation accuracies of chromosomal breaks induced in the suicide deletion assay. Ligation accuracy is determined by dividing the number of red colonies to the total number of surviving cells times 100.

The HO survival assays do not measure the mutagenic ligation events occurring at the chromosomal breaks. Therefore we employed the suicide deletion assay to measure the accuracy of the chromosomal repair in the mutants [48]. In this assay the *ADE2* gene is disrupted by the insertion of a cassette containing the galactose-inducible *I-SceI* gene and the selectable *URA3* gene between two flanking I-SceI sites (Figure 4A) [48]. Induction of I-SceI causes removal of the *I-SceI-URA3* cassette and, if ligation is accurate, it restores a functional *ADE2* gene, which renders the resultant colonies white. If NHEJ is not accurate, mutagenesis of *ADE2* results in red colonies. We first tested sensitivity of the mutants to the I-SceI induced chromosomal breaks. As shown in Figure 4B, the *sub1Δ* mutant has a slightly increased survival compared to wild type, while the *yku70Δ* mutant and the double mutant have similar reduced survival rates. Further analysis of the surviving cells reveals comparable frequencies of accurate repair when wild type and the *sub1* mutant are compared (Figure 4C). In contrast, repair accuracy is much lower in the *yku70Δ* mutant (Figure 4C), as reported previously [21]. The double mutant exhibits a high rate of mutagenic ligation similar to the *yku70Δ* mutant, suggesting deletion of *YKU70* results a dominant mutagenic phenotype in chromosomal NHEJ repair.

## Discussion

We have shown that *SUB1* is required for NHEJ repair of DNA breaks in plasmids, but not in chromosomes. The fact that deletion of *SUB1* does not reduce accuracy of NHEJ indicates that Sub1 functions differently from the *KU* complex, which is required for accurate ligations. In fact, the dispensable role of Sub1 in chromosomal repair suggests that Sub1 is not a core component of NHEJ, since *KU* and other NHEJ factors, but not Sub1, are absolutely required for NHEJ repair of chromosomal breaks.

Previously a microarray-based screen has been performed by Ooi *et al* to identify new genes that are required for repairing plasmid DSBs [22]. While the screen has been proven useful, successfully identifying *NEJ1* as a novel component in NHEJ, *SUB1* was not reported. However, several mutants including *rad9Δ*, *rad17 Δ*, *rad24 Δ*, and *srs2Δ* that are known to have reduced NHEJ efficiencies were also not identified in the microarray-based screen [22]. Furthermore, the authors reported that 13% of haploid mutants were not analyzed due to high signal noise. Thus, it is possible that *SUB1* was missed in this screen.

It is interesting to note that the *sub1Δ* mutant, unlike the *yku70Δ* mutant, is deficient in repairing the blunt-end DSB in plasmid DNA. It has long been found that blunt-end DSB is repaired at a low efficiency and that knockout mutations of Yku70 or Yku80 can increase the efficiency of repair [21], [49]. Furthermore, Westmoreland *et al* found that yeast cells inefficiently survive PvuII-induced chromosomal breaks and this survival is not affected by deletion of *RAD52* or *DNL4* [51], suggesting repair of blunt-end DSBs is independent of HR or canonical NHEJ. It remains elusive how blunt-end DSBs are repaired independently of KU. Our results show that deletion of *SUB1* reduces the efficiency of the already-inefficient repair of blunt-end DSBs. However, in the absence of *YKU70*, repair of blunt-end DSBs is higher than wild type, regardless of whether Sub1 is functional or not. We speculate that when repair is channeled into the KU-independent pathway, Sub1 no longer plays a role.

Given the pronounced effect of *SUB1* on repair all types of DSBs in plasmids, it is surprising to find that Sub1 is dispensable for repair of chromosomal breaks, whether they are induced by HO or I-SceI. While the reasons remain to be determined, this is reminiscent of the work by Batta *et al.* They found that PC4 enhances DNA ligation *in vitro* but human cells with PC4 knocked down are not sensitive to DSB inducing reagents [41]. In this study it was shown by atomic force microscopy that PC4 has activity that bridges DNA ends [41]. Thus the proposed model has been that PC4 facilitates ligation of plasmid DSBs by bridging the free DNA ends. Indeed, in yeast the ends of chromosomal breaks have been shown to be held in place by chromatin structures after chromosomal breaks are induced [53]–[55], whereas the ends of plasmid DNA are produced prior to transformation and therefore not held in close proximity. This distinct difference between ends of chromosomal breaks and plasmid breaks may underlie the differential requirement of *SUB1* in repair of plasmid DSBs versus chromosomal DSBs.

We do not rule out other possible reasons that may explain the differential requirement of *SUB1* in repairing plasmid DNA and chromosomal DNA. For example, as a DNA binding protein, Sub1 could potentially affect DNA resection. DNA end resection can have different outcomes in long chromosomes versus short DNA of plasmids: resection at both ends of the plasmid will destroy it, resulting in an apparent low repair efficiency. Alternatively, the effects of Sub1 can be indirect as Sub1 is a transcription factor. However, the plausible targets of transcriptional regulation are not expected to be any known NHEJ factors that are required for repair of chromosomal breaks, since the consequences of *SUB1* deficiency is different and unique compared to the consequences due to loss of known NHEJ factors.

The plasmid repair assay has been widely used to test the ability of the cells to repair DNA breaks [15], [56]. Often the conclusions are extended to implicate the ability of the cells to repair chromosomal breaks. Here we provide an example that such implications may not be warranted, since the strong genetic requirement for Sub1 in NHEJ of transformed plasmid DNA does not extend to repair of endonuclease induced chromosomal double-strand DNA breaks. In summary, our results clearly demonstrate that repair of transformed DNA and chromosomal or chromatin associated DNA is quite different in their requirement for Sub1. While many of the genetic requirements are identical, the ability to genetically separate the two methods of assessing NHEJ clearly demonstrates that the two substrates are not repaired in an identical manner.
